# The DNA Copy Number of Human Endogenous Retrovirus-W (MSRV-Type) Is Increased in Multiple Sclerosis Patients and Is Influenced by Gender and Disease Severity

**DOI:** 10.1371/journal.pone.0053623

**Published:** 2013-01-07

**Authors:** Marta Garcia-Montojo, María Dominguez-Mozo, Ana Arias-Leal, Ángel Garcia-Martinez, Virginia De las Heras, Ignacio Casanova, Raphaël Faucard, Nadège Gehin, Alexandra Madeira, Rafael Arroyo, François Curtin, Roberto Alvarez-Lafuente, Hervé Perron

**Affiliations:** 1 Multiple Sclerosis Unit, Hospital Clínico San Carlos, Instituto de Investigación Sanitaria del Hospital Clínico San Carlos, Madrid, Spain; 2 Geneuro Innovation, Lyon, France; Charite Universitätsmedizin, Germany

## Abstract

**Background:**

Multiple Sclerosis is an autoimmune disease more prevalent in women than in men. Multiple Sclerosis Associated Retrovirus element (MSRV) is a member of type-W endogenous retrovirus family (HERV-W), known to be associated to MS. Most HERVs are unable to replicate but MSRV expression associated with reverse-transcriptase activity in MS would explain reported DNA copy number increase in MS patients. A potential link between HERV-W copies on chromosome X and gender differential prevalence has been suggested. The present study addresses MSRV-type DNA load in relation with the gender differences and clinical status in MS and healthy controls.

**Results:**

178 MS patients (62.9% women) and 124 controls (56.5% women) were included. MSRV *env* load (copies/pg of DNA) was analyzed by real time qPCR with specific primers and probe for its *env* gene, in DNA from peripheral blood mononuclear cells (PBMCs). MSRV load was more elevated in MS patients than in controls (p = 4.15e-7). MS women presented higher MSRV load than control women (p = 0.009) and MS men also had higher load than control men (p = 2.77e-6). Besides, women had higher levels than men, both among patients (p = 0.007) and controls (p = 1.24e-6). Concordantly, EDSS and MSSS scores were higher among female patients with an elevated MSRV load (p = 0.03 and p = 0.04, respectively).

**Conclusions:**

MSRV increases its copy number in PBMC of MS patients and particularly in women with high clinical scores. This may explain causes underlying the higher prevalence of MS in women. The association with the clinical severity calls for further investigations on MSRV load in PBMCs as a biomarker for MS.

## Introduction

Multiple Sclerosis Associated Retrovirus (MSRV) is a member of type-W Human Endogenous Retroviruses (HERV-W), an HERV family suggested to be involved in Multiple Sclerosis (MS) pathogenesis [1]. The study of the relationship between HERVs and MS began in 1989, when retroviral particles were discovered in cultures of leptomeningeal cells [2] and monocytes [3] from MS patients. In independent epidemiological studies, an association between MSRV/HERV-W and MS diagnosis [1], [4] and prognosis [5], [6] has been confirmed.

Other HERV families have been also studied in relation with MS: a *HERV-K18* haplotype is associated with MS susceptibility [7] and increased levels of *HERV-H* members RNA and antibodies towards peptides derived from *HERV-H* DNA have been found in MS patients compared to controls [8], [9].

We have focused on HERV-W because (i) MSRV causes a T cell-mediated neuropathology *in vivo* [10], (ii) MSRV/HERV-W envelope protein induces a potent activation of innate immunity and subsequent release of pro-inflammatory cytokines, through Toll-like receptor (TLR-4) [11] and (iii) acts as superantigen, producing polyclonal T-lymphocyte activation [12]. Moreover, HERV-W env protein can induce oligodendrocyte toxicity inflammation-mediated [13]. Along the evolution, most of the HERV proviruses have undergone extensive deletions and mutations and are present in the genome as defective copies unable to replicate. However, HERVs can be transactivated by other viruses also associated with MS pathogenesis as Herpesviruses: Epstein-Barr (EBV) [14], Human Herpesvirus-1 (HSV1) [15] or Human Herpesvirus-6 (HHV6) [16].

Further linking this HERV-W expression with MS, successive studies have evidenced that MSRV env protein was found in the serum of 73% of MS patients and not in controls [1] and MSRV*/*HERV-W *pol* and *env* RNAs were significantly elevated in autopsied brain tissue and peripheral blood mononuclear cells (PBMCs) from MS patients versus controls [17], [18].

The role of gender in the natural history of MS has different aspects, but the best known fact is the higher prevalence of the disease among women. The sex ratio continues to intrigue researchers and it has been shown that this female predominance has even increased over past decades [19], [20]. However, gender issues in MS expand beyond the scope of sex ratio [21]. Gender has an impact on various aspects of MS, including age of onset [22], “parent-of-origin” effect on susceptibility [23], [24] and risk for relatives of MS patients [25].

A potential link between MSRV and the gender differences observed in MS that involves the HERV-W copies on chromosome X has been suggested [26]. The exact genomic origin of MSRV is unknown, however, analysis of MSRV-type *env* sequences revealed that some of them could originate from transcripts (in some instances recombined) of defective HERV-W elements [27]; among all the HERV-W locations, the copy on chromosome Xq22.3 has been found to encompass a locus (ERVWE2) encoding a truncated envelope protein, which however cannot explain by itself a full-length protein detection nor detection of virions associated with reverse transcriptase activity [28].

Two components of the HERV-W family have been shown to display immunopathogenic activity that could be relevant for MS: MSRV and *ERVWE1* (from HERV-W7q copy, encoding syncytin-1). *ERVWE1* and MSRV *env* are closely related, but they have a 12-nucleotide difference in the trans-membrane moiety [29], [30]. A discriminatory PCR study has recently shown that only MSRV*-*type *env* and not *ERVWE1* presents an increased expression in PBMCs of multiple sclerosis patients [29]. Besides, the HERV-W7q copy comprising the *ERVWE1* locus has defective *pol* and *gag* genes so it is unlikely to be involved in the genesis of supplementary DNA copies.

In the present study the levels of MSRV-type DNA sequences in PBMCs from MS patients and controls were measured by specific quantitative PCR (qPCR) analysis. The results were analyzed in relation with gender differences and MS clinical scores.

## Materials and Methods

### Ethics Statement

Informed consent was signed by all the individuals and the study was approved by the Ethics Committee of the Hospital Clinico San Carlos.

### Patients and Controls

178 MS patients (62.9% women) and 124 controls (ethnically, age and sex matched healthy blood donors; 56.5% women) were included from a single center (Hospital Clinico San Carlos, Madrid). All the patients but 10 were under treatment. All of them were Caucasian, with European ancestors. MS diagnosis was established according to McDonald’s criteria [31]. No first or second degree relatives with autoimmune diseases were reported by the control subjects. Clinical and demographic characteristics are enclosed in Table 1. Clinical variables collected included MS course, Expanded Disability Status Scale (EDSS) score [32], Multiple Sclerosis Severity Scale (MSSS) score [33], years of evolution of the disease and number of relapses within the last two years.

**Table 1:** Clinical and demographic characteristics of MS patients and controls, stratified by sex.

Characteristics	Patients				Controls				p-value
	Total	Men	Women	p-value	Total	Men	Women	p-value	
n (% of total)	178 (100)	67 (37.4)	112 (62.6)	–	124 (100)	54 (43.5)	70 (56.5)	–	n.s*
Age (years) (Mean ± SD)	36.7±10.6	41.4±10.4	39.3±9.5	n.s*	40.1±9.9	38.8±9.1	41.0±11.6	n.s*	n.s
RR (n (%))	112 (64.4)	40 (61.5)	72 (66.1)	n.s*	–	–	–	–	–
SP (n (%))	38 (21.8)	14 (21.5)	24 (22.0)	n.s*	–	–	–	–	–
PP (n (%))	24 (13.8)	11 (16.9)	13 (11.9)	n.s*	–	–	–	–	–
Disease duration (years)	8.1±5.6	8.6±6.6	7.7±4.9	n.s*	–	–	–	–	–
(Mean ± SD)									
Number of relapses in the lasttwo years (Mean ± SD)	0.83±1.12	0.63±0.72	0.94±1.29	n.s*					
									
Current EDSS score (Mean ± SD)	2.93±2.4	2.85±2.24	2.97±2.50	n.s*	–	–	–	–	–
Current MSSS score (Mean ± SD)	3.97±3.09	3.79±2.79	4.08±3.16	n.s*					

* n.s: Not statistically significant.

### Determination of Specificity and Efficiency of the PCR Assay

To determine the specificity of the MSRV*-type* set of primers and probe [29], real time PCR assays were performed on serial dilutions of MSRV *env* and s*yncytin-1* plasmids (available at Geneuro S.A, Geneva, Switzerland) confirming that it was able to detect MSRV *env* (GenBank accession number: AF331500) but not s*yncytin-1* (Accession number: AC000064). A standard curve of serial dilutions of human genomic DNA was used to determine the efficiencies of MSRV *env* and *RNAse P* PCR assays (Supporting information: Figures S1 and S2). Efficiency of MSRV *env* assay was determined also by a standard curve of MSRV plasmid (Supporting information: Figure S3). Both MSRV standard curves (plasmid and genomic DNA) had similar slopes. The slopes were −3.4 for RNAse P and −3.2 for MSRV.

### *In silico* Analysis

To determine the number of copies of HERV-W MSRV*-type* potentially detected in the human genome the sequences of the primers and probe [29] were aligned with the BLASTN tool available at Ensembl [34].

### MSRV-*type* DNA Copy Number Quantification

PBMCs were obtained from fresh whole blood by centrifugation of CPT tubes (Becton Dickinson, Meylan, France). DNA was extracted from PBMCs with the Qiamp DNA Blood Mini kit (Qiagen, Valencia, CA, USA) following manufacturer’s instructions. DNA quality and concentration was assessed by spectrophotometer and sample volumes were adjusted to 10 ng/ul, corresponding to 50 ng of DNA per PCR reaction. Each sample was analyzed in duplicate by real time PCR with a set of primers and probe to detect specifically MSRV*-type env* [29] and RNAse P (Applied Biosystems). *RNAse P* is a single-copy gene and it was used to normalize the results with the DNA load per reaction, avoiding potential errors with pipetting.

Each round included an interplate calibrator (I.P.C), consisting of human DNA with a known copy number of MSRV *env* DNA, and a negative control; they were analyzed for both genes. The assays for the detection of RNAse P and MSRV*-type env* were considered acceptable in each sample when: 1) No amplification was detected in the negative controls; 2) Ct of RNAse P was lower than Mean +2*S.D of Ct_RNAse P_ of all samples; 3) the duplicates of each sample had less than 5% of variability.

Genex® (MultiD analyses AB, Sweden) software was used to normalize sample Cts according to the efficiencies of each assay and also to the Cts of the I.P.C in each round.

The exact number of copies of MSRV*-type* was determined by interpolation of Cts in a standard curve of serial dilutions of MSRV plasmid (available at Geneuro S.A); the exact number of RNAse P copies per reaction was determined by interpolation of Cts in a standard curve of genomic DNA. There is only one copy of RNAse P per haploid genome and the mass of the haploid genome is 3.5 pg [35], thus interpolating the Cts of RNAse P in the standard curve of genomic DNA the exact quantity of DNA loaded in the reaction is obtained. Results are expressed as MSRV*-type env* copies/pg of DNA.

### Statistical Analysis

Statistical analyses were performed with SPSS 15.0. Chi-Square test was used to compare qualitative variables. As MSRV load (MSRV*-type env* copies/pg of DNA) was not normally distributed (Kolmogorov-Smirnov test) a log_10_ transformation of the data was made. Data are expressed as mean [95% confidence interval for the mean]. ANOVA test was used for comparison of groups.

To compare patients with a high MSRV DNA load with the rest, a cut-off was calculated: Cut-Off = Mean +2*S.D (log_10_ MSRV*-type env* copies/pg DNA)_Blood Donors_; (Cut-Off = 2.22, corresponding to 165 copies/pg of DNA) thus, representing significantly increased values from the normal range in a healthy population. Clinical variables were compared between patients with a MSRV*-type* load above and below the cut-off.

Statistically significant differences were considered when p<0.05.

## Results and Discussion

**Figure 1:**
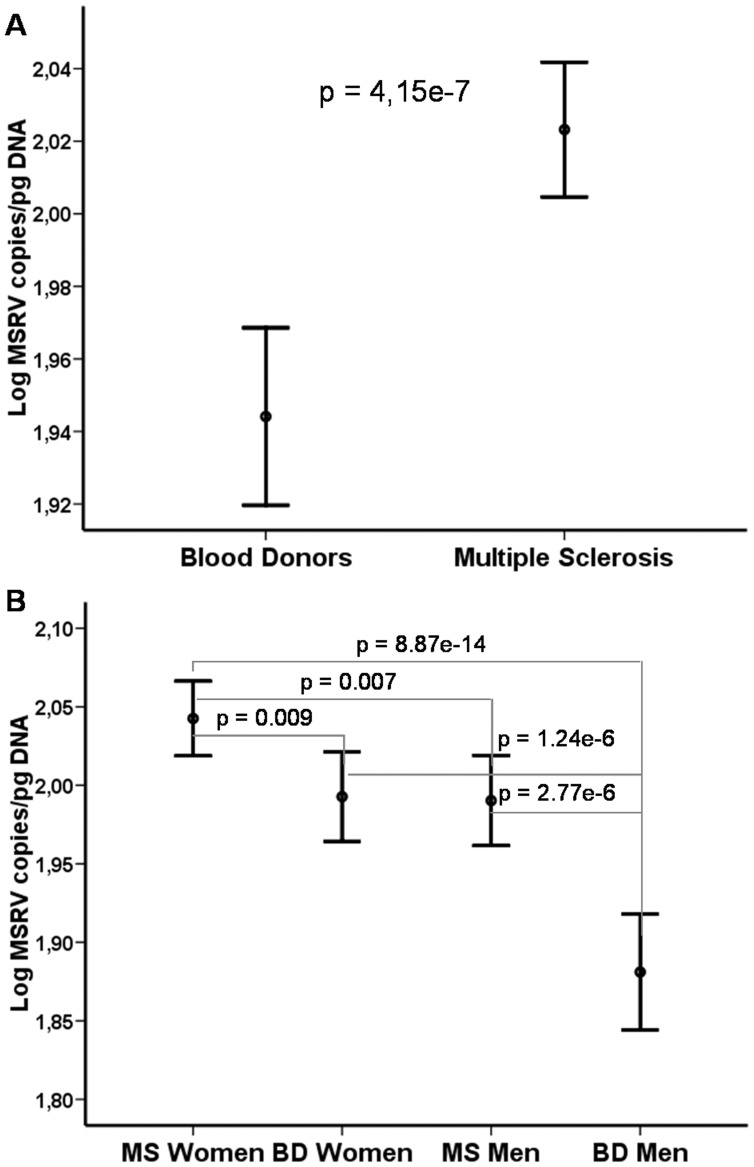
MSRV DNA load MS diagnosis and sex. **A)** MSRV DNA load is more elevated in PBMC of MS patients (n = 178) than in controls (n = 124) (ANOVA; p = 4.15e-7). B**)** MSRV DNA load is more elevated in MS women (n = 112) than in control women (n = 70) (ANOVA; p = 0.009), control men (n = 54) (ANOVA; p = 8.87e-14) and MS men (n = 66) (ANOVA; p = 0.007). MSRV DNA load is more elevated in control women (n = 70) than in control men (n = 54) (ANOVA; p = 1.24e-6). Points represent the mean and bars represent 95% Confidence Interval of Mean. MSRV DNA load represents MSRV copies/pg of DNA.

The analyses by qPCR revealed that MSRV-type DNA copy number was more elevated in PBMCs of MS patients (Mean = 110 [104–115] MSRV copies/pg of DNA) than in controls (Mean = 92 [87–97] MSRV copies/pg of DNA) (ANOVA; p = 4.15e-7) (Figure 1A). Beyond a strong significance of these data, since characterizing copy number variation in the genomic DNA of human cells from *ex vivo* samples, the presently improved method replicates results of previous studies [1], [29]. In the original study showing an increase in MSRV DNA levels in MS patients (n = 8) compared to controls (n = 6) [29] authors measured the relative levels of MSRV DNA. It was found that MSRV *env* DNA load, but not that of Syncytin gene (*ERVWE1*), was increased in PBMCs of MS patients. In the present study this result is replicated in a higher number of patients (n = 178) and controls (n = 124). The present method includes normalization with a single-copy gene (*RNAse P*), verification of similar efficiencies for target and normalization gene assays and interplate calibration [36]. Moreover, our data were systematically analyzed with a real-time PCR software (Genex®, MultiD analyses AB, Sweden). This method therefore avoids biases such as differences in DNA load or integrity, or resulting from interplate variability.

The stratification by group and sex (Figure 1B) revealed that MSRV-*env* DNA load varies with the disease status of MS patients and with the gender. When considering differences between patients and controls, MS women (n = 112; Mean = 115 [108–122] MSRV copies/pg of DNA) had increased genomic load compared to control women (n = 70; Mean = 101 [95–108] MSRV copies/pg of DNA; ANOVA; p = 0.009). Similarly, MS men (n = 66; Mean = 101 [94–108] MSRV copies/pg of DNA) also had increased MSRV-*env* copies compared to control men (n = 54; Mean = 79 [72–86] MSRV copies/pg of DNA; ANOVA; p = 2.77e-6). When considering gender among MS patients, MSRV DNA copy number was elevated in women compared to men (ANOVA; p = 0.007) but, interestingly, among controls as well (ANOVA; p = 1.24e-6).

Elsewhere, determining the exact number of MSRV-type copies per genome is difficult both *in silico* and *ex-vivo*. Alignment of our primers and probe sequences on Ensembl database resulted in a potential detection of 39 copies spread in 17 chromosomes: 1, 2, 3, 5, 6, 7, 8, 9, 10, 11, 12, 14, 15, 19, 20, 21 and X. However, this number can change according to the specificity of the BLAST. On the other hand, the number of MSRV-type copies can be different from one cell to another in MS patients, since expression of MSRV-env together with reverse transcriptase and retroviral particles is limited to a subpopulation of cells [37]. In fact, as shown by Brudek et al. [38], only a percentage of monocytes and B-cells displayed an increased expression of HERV-W in active MS patients compared to controls. For these reasons, we have expressed our results in MSRV*-type* copies per pg of DNA, and not per cell.

The different loads of MSRV *env* DNA between men and women among control individuals could be related to the HERV-W *env* copies present in chromosome X (at least two per chromosome, as detected after alignment on Ensembl database), since they would be detected in duplicate in women.

The more elevated MSRV load in MS patients’ DNA may then indicate that, in MS, the MSRV pathogenic copy can retrotranpose in PBMCs with active MSRV reverse-trancriptase [39] and, eventually integrase [30] through recombination/integration events. However, to determine if these new copies are actually retro-inserted, other complementary methods should be used as FISH or Southern Blotting, since reverse-transcribed copies may also remain episomal in cell nucleus.

**Figure 2:**
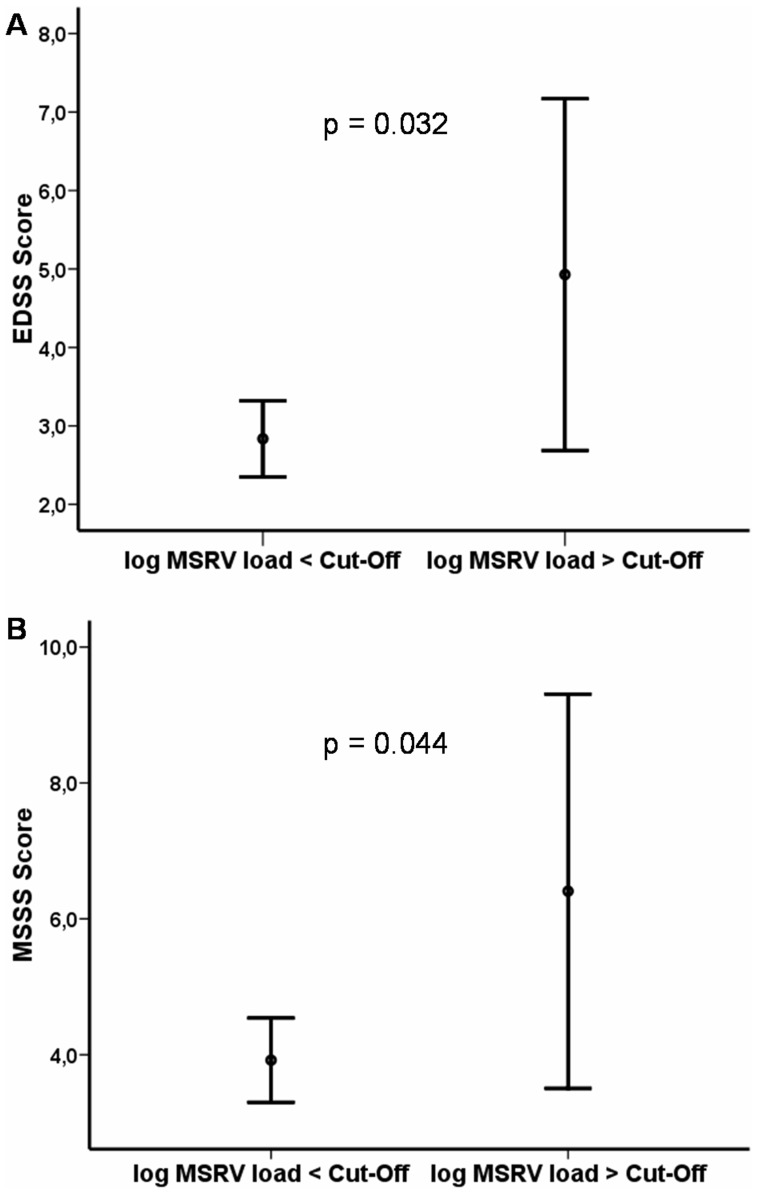
MSRV load and clinical evolution in women with MS. **A)** The EDSS Score is higher among patients with a MSRV DNA load above the Cut-Off* (Cut-Off = 2.22, corresponding to 165 copies/pg of DNA) (n = 7) than below (n = 101). ANOVA; p = 0.032. **B)** MSSS score is higher among patients with a MSRV DNA load above the Cut-Off*(n = 7) than below (n = 99). ANOVA; p = 0.044. Points represent the mean and bars represent 95% Confidence Interval of Mean. MSRV load represents MSRV copies/pg of DNA. *Cut-Off = Mean _log10_ _MSRV DNA_ _Controls_ +2*S.D _log10 MSRV DNA_ _Controls;_ This represents the threshold above which the values are no longer within the range of the normal population.

Several groups have shown increased levels of MSRV-type or total HERV-W RNA transcription in serum, brain or PBMCs of MS patients compared to controls [17], [40], [41]. The reverse-transcription and eventual retrotransposition of mRNA would favor an increase in its genomic copy number, thus detected among the whole PBMCs genomic DNA from present samples (comprising altogether Monocytes, NK cells, T- and B-Lymphocytes). Moreover, retrotransposition can lead to the formation of retrogenes which can be transcribed and translated [42]. If such retrotransposed HERV-W*/*MSRV *env* copies were constitutively expressed, it would obviously aggravate the disease course. Indeed, regarding clinical evolution, EDSS (Figure 2A) and MSSS (Figure 2B) scores in women were higher among patients with a MSRV load above the Cut-Off of normal population (Cut-Off = 2.22, corresponding to 165 copies/pg of DNA; EDSS = 4.92 [2.68–7.16]; Mean MSSS = 6.40 [3.50–9.30] than among patients with a load below this normal threshold (Mean EDSS = 2.83 [2.35–3.32]; Mean MSSS = 3.92 [3.29–4.54]; ANOVA; p = 0.032 and p = 0.044, respectively).

Although this result is interesting, the practical use of MSRV-type *env* DNA copy number as a biomarker for MS should be confirmed through longitudinal studies for comparison of DNA levels during different periods of the disease, as well as pre- and post-treatment.

Other groups have supported a potential usefulness of HERV-W*/*MSRV as MS biomarker. In longitudinal evaluations of MS patients during interferon-beta therapy MSRV RNA load in the blood was directly related to MS duration and it fell after 3 months of interferon-beta therapy [43]; besides, HERV-W protein detection by anti-envelope antibodies tended to decrease as a consequence of efficient IFN-beta therapy [44].

At this time there are no experimental proofs of *de novo* insertions of HERV-W. However, insertional polymorphism of another HERV family, the HERV-K, has been evidenced [45], [46]. Moreover, a retroviral sequence highly similar to MSRV was identified by Representational Difference Analysis (RDA) in three pairs of monozygotic twins discordant for schizophrenia [47] and the authors proposed that retroviral sequences transposing during fetal growth may alter neurodevelopmental genes and cause the disease.

The increased expression of HERV-W env and pol (that contains the highly conserved reverse transcriptase and integrase domains) in MS [30] and the higher reverse-transcriptase activity found in MS patients [48] compared to controls strongly suggest that retro-integration of endogenous retroviral sequences could be an ongoing phenomenon occurring in some pathological conditions, leading to the increased levels of HERV DNA observed in MS. The reintegration of these sequences could also be mediated through long interspersed nuclear elements (LINEs), retroposons that have retained their ability to retrotranspose [49]. Indeed, it is has been shown that HERV-W processed pseudogenes have a strong preference for the insertion motif of long interspersed nuclear element (LINE) retrotransposons, suggesting that HERV-W processed pseudogenes arose by multiple and independent LINE-mediated retrotransposition of retroviral mRNA [50].

The more elevated MSRV*-type env* load in MS women would be compatible with the hypothesis of a genomic origin of MSRV in chromosome X harboring a complete MSRV provirus, most probably in a -yet unidentified- subgroup of individuals. This could possibly involve the ERVWE2 locus, though this locus is partially defective in the normal population, has nonetheless retained partial coding capacity and can produce an N-terminally truncated Env protein *in vitro* [28]. Alternatively, MSRV expression could result from the simultaneous presence of active proviral “mosaic” genes with orfs permitting a complete set to be expressed from such dispersed elements [30], [51].

This might be relevant, at least in some individuals, given the significant excess of retrogenes originating from the X chromosome in the human genome [52], [53]. HERV-W molecular features, in conjunction with X chromosome ratio, could thus underlie the higher prevalence of the MS in women than in men (ratio 2–3∶1) [54].

The higher prevalence of autoimmune diseases in women is a well known phenomenon. A putative localization of the genomic origin of MSRV in chromosome X would support the critical involvement of X chromosome gene products in the female predisposition to MS. In fact, sex-based differences in MS have been suggested to be due to defects in X chromosomes, including skewed X chromosome inactivation or reactivation of inactivated chromosome through loss of epigenetic control in women [55]. Reactivation of an inactivated X chromosome in women can thus result in an over-expression of certain X-linked genes affecting immune function [56].

These mechanisms have to be more deeply studied in order to clarify the source of such increase in MSRV-type copy number. On the other hand, other sources of variability as Microhomology-Mediated Break-Induced Replication that can lead to copy number variation cannot be ruled out [57].

From the present data available in the domain and our study results, it can be concluded that MSRV is likely to increase its DNA copy number in MS patients through reverse-transcription in PBMC and, possibly, with chromosomal retrotransposition. Its more elevated proviral load in women than in men could underlie gender differences in MS. Finally, the association with the clinical severity supports further investigations on the use of MSRV genomic load as a disease biomarker for MS.

## Supporting Information

Figure S1 **Standard Curve of MSRV env made of serial dilutions of genomic DNA.**
(PDF)

Figure S2 **Standard Curve of RNAse P made of serial dilutions of genomic DNA.**
(PDF)

Figure S3 **Standard Curve of MSRV env made of serial dilutions of MSRV env plasmid.**
(PDF)

## References

[1] Perron H, Germi R, Bernard C, Garcia-Montojo M, Deluen C, et al.. (2012) Human endogenous retrovirus type W envelope expression in blood and brain cells provides new insights into multiple sclerosis disease. Mult Scler. 10.1177/1352458512441381 PMC3573672 22457345

[2] Perron H, Geny C, Laurent A, Mouriquand C, Pellat J, et al (1989) Leptomeningeal cell line from multiple sclerosis with reverse transcriptase activity and viral particles. Res Virol 140: 551–561. 2482522 10.1016/s0923-2516(89)80141-4

[3] Perron H, Lalande B, Gratacap B, Laurent A, Genoulaz O, et al (1991) Isolation of retrovirus from patients with multiple sclerosis. Lancet 337: 862–863. 1707471 10.1016/0140-6736(91)92579-q

[4] Dolei A, Serra C, Mameli G, Pugliatti M, Sechi G, et al (2002) Multiple sclerosis-associated retrovirus (MSRV) in Sardinian MS patients. Neurology 58: 471–473. 11839854 10.1212/wnl.58.3.471

[5] Sotgiu S, Serra C, Mameli G, Pugliatti M, Rosati G, et al (2002) Multiple sclerosis-associated retrovirus and MS prognosis: an observational study. Neurology 59: 1071–1073. 12370465 10.1212/wnl.59.7.1071

[6] Sotgiu S, Arru G, Mameli G, Serra C, Pugliatti M, et al (2006) Multiple sclerosis-associated retrovirus in early multiple sclerosis: a six-year follow-up of a Sardinian cohort. Mult Scler 12: 698–703. 17262996 10.1177/1352458506070773

[7] Tai A, O’Reilly E, Alroy K, Simon K, Munger K, et al (2008) Human endogenous retrovirus-K18 Env as a risk factor in multiple sclerosis. Mult Scler 14: 1175–1180. 18701576 10.1177/1352458508094641 PMC2754175

[8] Christensen T, Dissing SP, Riemann H, Hansen HJ, Munch M, et al (2000) Molecular characterization of HERV-H variants associated with multiple sclerosis. Acta Neurol Scand 101: 229–238. 10770518 10.1034/j.1600-0404.2000.101004229.x

[9] Laska MJ, Brudek T, Nissen KK, Christensen T, Moller-Larsen A, et al (2012) Expression of HERV-Fc1, a human endogenous retrovirus, is increased in patients with active multiple sclerosis. J Virol 86: 3713–3722. 22278236 10.1128/JVI.06723-11 PMC3302483

[10] Firouzi R, Rolland A, Michel M, Jouvin-Marche E, Hauw JJ, et al (2003) Multiple sclerosis-associated retrovirus particles cause T lymphocyte-dependent death with brain hemorrhage in humanized SCID mice model. J Neurovirol 9: 79–93. 12587071 10.1080/13550280390173328

[11] Rolland A, Jouvin-Marche E, Viret C, Faure M, Perron H, et al (2006) The envelope protein of a human endogenous retrovirus-W family activates innate immunity through CD14/TLR4 and promotes Th1-like responses. J Immunol 176: 7636–7644. 16751411 10.4049/jimmunol.176.12.7636

[12] Perron H, Jouvin-Marche E, Michel M, Ounanian-Paraz A, Camelo S, et al (2001) Multiple sclerosis retrovirus particles and recombinant envelope trigger an abnormal immune response in vitro, by inducing polyclonal Vbeta16 T-lymphocyte activation. Virology 287: 321–332. 11531410 10.1006/viro.2001.1045

[13] Antony JM, van MG, Opii W, Butterfield DA, Mallet F, et al (2004) Human endogenous retrovirus glycoprotein-mediated induction of redox reactants causes oligodendrocyte death and demyelination. Nat Neurosci 7: 1088–1095. 15452578 10.1038/nn1319

[14] Sutkowski N, Conrad B, Thorley-Lawson DA, Huber BT (2001) Epstein-Barr virus transactivates the human endogenous retrovirus HERV-K18 that encodes a superantigen. Immunity 15: 579–589. 11672540 10.1016/s1074-7613(01)00210-2

[15] Ruprecht K, Obojes K, Wengel V, Gronen F, Kim KS, et al (2006) Regulation of human endogenous retrovirus W protein expression by herpes simplex virus type 1: implications for multiple sclerosis. J Neurovirol 12: 65–71. 16595376 10.1080/13550280600614973

[16] Tai AK, Luka J, Ablashi D, Huber BT (2009) HHV-6A infection induces expression of HERV-K18-encoded superantigen. J Clin Virol 46: 47–48. 19505843 10.1016/j.jcv.2009.05.019

[17] Mameli G, Astone V, Arru G, Marconi S, Lovato L, et al (2007) Brains and peripheral blood mononuclear cells of multiple sclerosis (MS) patients hyperexpress MS-associated retrovirus/HERV-W endogenous retrovirus, but not Human herpesvirus 6. J Gen Virol 88: 264–274. 17170460 10.1099/vir.0.81890-0

[18] Antony JM, Izad M, Bar-Or A, Warren KG, Vodjgani M, et al (2006) Quantitative analysis of human endogenous retrovirus-W env in neuroinflammatory diseases. AIDS Res Hum Retroviruses 22: 1253–1259. 17209768 10.1089/aid.2006.22.1253

[19] Orton SM, Herrera BM, Yee IM, Valdar W, Ramagopalan SV, et al (2006) Sex ratio of multiple sclerosis in Canada: a longitudinal study. Lancet Neurol 5: 932–936. 17052660 10.1016/S1474-4422(06)70581-6

[20] Krokki O, Bloigu R, Reunanen M, Remes AM (2011) Increasing incidence of multiple sclerosis in women in Northern Finland. Mult Scler 17: 133–138. 20935028 10.1177/1352458510384012

[21] Sadovnick AD (2009) European Charcot Foundation Lecture: the natural history of multiple sclerosis and gender. J Neurol Sci 286: 1–5. 19782378 10.1016/j.jns.2009.09.005

[22] Cossburn M, Ingram G, Hirst C, Ben-Shlomo Y, Pickersgill TP, et al (2012) Age at onset as a determinant of presenting phenotype and initial relapse recovery in multiple sclerosis. Mult Scler 18: 45–54. 21865412 10.1177/1352458511417479

[23] Ramagopalan SV, Yee IM, Dyment DA, Orton SM, Marrie RA, et al (2009) Parent-of-origin effect in multiple sclerosis: observations from interracial matings. Neurology 73: 602–605. 19515994 10.1212/WNL.0b013e3181af33cf PMC2830902

[24] Herrera BM, Ramagopalan SV, Lincoln MR, Orton SM, Chao MJ, et al (2008) Parent-of-origin effects in MS: observations from avuncular pairs. Neurology 71: 799–803. 18480463 10.1212/01.wnl.0000312377.50395.00

[25] Ebers GC, Sadovnick AD, Dyment DA, Yee IM, Willer CJ, et al (2004) Parent-of-origin effect in multiple sclerosis: observations in half-siblings. Lancet 363: 1773–1774. 15172777 10.1016/S0140-6736(04)16304-6

[26] Perron H, Bernard C, Bertrand JB, Lang AB, Popa I, et al (2009) Endogenous retroviral genes, Herpesviruses and gender in Multiple Sclerosis. J Neurol Sci 286: 65–72. 19447411 10.1016/j.jns.2009.04.034

[27] Laufer G, Mayer J, Mueller BF, Mueller-Lantzsch N, Ruprecht K (2009) Analysis of transcribed human endogenous retrovirus W env loci clarifies the origin of multiple sclerosis-associated retrovirus env sequences. Retrovirology 6: 37. 19368703 10.1186/1742-4690-6-37 PMC2672075

[28] Roebke C, Wahl S, Laufer G, Stadelmann C, Sauter M, et al (2010) An N-terminally truncated envelope protein encoded by a human endogenous retrovirus W locus on chromosome Xq22.3. Retrovirology 7: 69. 20735848 10.1186/1742-4690-7-69 PMC2936387

[29] Mameli G, Poddighe L, Astone V, Delogu G, Arru G, et al (2009) Novel reliable real-time PCR for differential detection of MSRVenv and syncytin-1 in RNA and DNA from patients with multiple sclerosis. J Virol Methods 161: 98–106. 19505508 10.1016/j.jviromet.2009.05.024

[30] Komurian-Pradel F, Paranhos-Baccala G, Bedin F, Ounanian-Paraz A, Sodoyer M, et al (1999) Molecular cloning and characterization of MSRV-related sequences associated with retrovirus-like particles. Virology 260: 1–9. 10405350 10.1006/viro.1999.9792

[31] McDonald WI, Compston A, Edan G, Goodkin D, Hartung HP, et al (2001) Recommended diagnostic criteria for multiple sclerosis: guidelines from the International Panel on the diagnosis of multiple sclerosis. Ann Neurol 50: 121–127. 11456302 10.1002/ana.1032

[32] Kurtzke J (1983) Rating neurologic impairment in multiple sclerosis: an expanded disability status scale (EDSS). Neurology 1983: 33–1444. 10.1212/wnl.33.11.1444 6685237

[33] Roxburgh RH, Seaman SR, Masterman T, Hensiek AE, Sawcer SJ, et al (2005) Multiple Sclerosis Severity Score: using disability and disease duration to rate disease severity. Neurology 64: 1144–1151. 15824338 10.1212/01.WNL.0000156155.19270.F8

[34] Ensembl Genome Browser website. Available: http://www.ensembl.org. Accessed 2012 Nov.

[35] Data base of Genome sizes website. Available: http://www.cbs.dtu.dk/databases/DOGS/index.html. Accessed 2012 Nov.

[36] Garson JA, Huggett JF, Bustin SA, Pfaffl MW, Benes V, et al (2009) Unreliable real-time PCR analysis of human endogenous retrovirus-W (HERV-W) RNA expression and DNA copy number in multiple sclerosis. AIDS Res Hum Retroviruses 25: 377–378. 19292592 10.1089/aid.2008.0270

[37] Perron H, Lalande B, Gratacap B, Laurent A, Genoulaz O, et al (1991) Isolation of retrovirus from patients with multiple sclerosis. Lancet 337: 862–863. 1707471 10.1016/0140-6736(91)92579-q

[38] Brudek T, Christensen T, Aagaard L, Petersen T, Hansen HJ, et al (2009) B cells and monocytes from patients with active multiple sclerosis exhibit increased surface expression of both HERV-H Env and HERV-W Env, accompanied by increased seroreactivity. Retrovirology 6: 104. 19917105 10.1186/1742-4690-6-104 PMC2780989

[39] Perron H, Firouzi R, Tuke P, Garson JA, Michel M, et al.. (1997) Cell cultures and associated retroviruses in multiple sclerosis. Collaborative Research Group on MS. Acta Neurol Scand Suppl 169: 22–31. 10.1111/j.1600-0404.1997.tb08146.x 9174637

[40] Nowak J, Januszkiewicz D, Pernak M, Liwen I, Zawada M, et al (2003) Multiple sclerosis-associated virus-related pol sequences found both in multiple sclerosis and healthy donors are more frequently expressed in multiple sclerosis patients. J Neurovirol 9: 112–117. 12587074 10.1080/13550280390173355

[41] de Villiers JN, Treurnicht FK, Warnich L, Carr J, van Rensburg SJ, et al (2006) Analysis of viral and genetic factors in South African patients with multiple sclerosis. Metab Brain Dis 21: 163–169. 16865539 10.1007/s11011-006-9016-3

[42] Feschotte C, Gilbert C (2012) Endogenous viruses: insights into viral evolution and impact on host biology. Nat Rev Genet 13: 283–296. 22421730 10.1038/nrg3199

[43] Mameli G, Serra C, Astone V, Castellazzi M, Poddighe L, et al (2008) Inhibition of multiple-sclerosis-associated retrovirus as biomarker of interferon therapy. J Neurovirol 14: 73–77. 18300077 10.1080/13550280701801107

[44] Petersen T, Moller-Larsen A, Thiel S, Brudek T, Hansen TK, et al (2009) Effects of interferon-beta therapy on innate and adaptive immune responses to the human endogenous retroviruses HERV-H and HERV-W, cytokine production, and the lectin complement activation pathway in multiple sclerosis. J Neuroimmunol 215: 108–116. 19766328 10.1016/j.jneuroim.2009.08.015

[45] Turner G, Barbulescu M, Su M, Jensen-Seaman MI, Kidd KK, et al (2001) Insertional polymorphisms of full-length endogenous retroviruses in humans. Curr Biol 11: 1531–1535. 11591322 10.1016/s0960-9822(01)00455-9

[46] Otowa T, Tochigi M, Rogers M, Umekage T, Kato N, et al (2006) Insertional polymorphism of endogenous retrovirus HERV-K115 in schizophrenia. Neurosci Lett 408: 226–229. 17000049 10.1016/j.neulet.2006.09.004

[47] Deb-Rinker P, Klempan TA, O’Reilly RL, Torrey EF, Singh SM (1999) Molecular characterization of a MSRV-like sequence identified by RDA from monozygotic twin pairs discordant for schizophrenia. Genomics 61: 133–144. 10534399 10.1006/geno.1999.5946

[48] Brudek T, Luhdorf P, Christensen T, Hansen HJ, Moller-Larsen A (2007) Activation of endogenous retrovirus reverse transcriptase in multiple sclerosis patient lymphocytes by inactivated HSV-1, HHV-6 and VZV. J Neuroimmunol 187: 147–155. 17493688 10.1016/j.jneuroim.2007.04.003

[49] Esnault C, Maestre J, Heidmann T (2000) Human LINE retrotransposons generate processed pseudogenes. Nat Genet 24: 363–367. 10742098 10.1038/74184

[50] Pavlicek A, Paces J, Elleder D, Hejnar J (2002) Processed pseudogenes of human endogenous retroviruses generated by LINEs: their integration, stability, and distribution. Genome Res 12: 391–399. 11875026 10.1101/gr.216902 PMC155283

[51] Perron H, Lang A (2010) The human endogenous retrovirus link between genes and environment in multiple sclerosis and in multifactorial diseases associating neuroinflammation. Clin Rev Allergy Immunol 39: 51–61. 19697163 10.1007/s12016-009-8170-x

[52] Wang PJ (2004) X chromosomes, retrogenes and their role in male reproduction. Trends Endocrinol Metab 15: 79–83. 15036254 10.1016/j.tem.2004.01.007

[53] Emerson JJ, Kaessmann H, Betran E, Long M (2004) Extensive gene traffic on the mammalian X chromosome. Science 303: 537–540. 14739461 10.1126/science.1090042

[54] Greer JM, McCombe PA (2011) Role of gender in multiple sclerosis: clinical effects and potential molecular mechanisms. J Neuroimmunol 234: 7–18. 21474189 10.1016/j.jneuroim.2011.03.003

[55] Pennell LM, Galligan CL, Fish EN (2012) Sex affects immunity. J Autoimmun 38: J282–J291. 22225601 10.1016/j.jaut.2011.11.013

[56] Lleo A, Battezzati PM, Selmi C, Gershwin ME, Podda M (2008) Is autoimmunity a matter of sex? Autoimmun Rev 7: 626–630. 18603021 10.1016/j.autrev.2008.06.009

[57] Hastings PJ, Ira G, Lupski JR (2009) A microhomology-mediated break-induced replication model for the origin of human copy number variation. PLoS Genet Jan 5(1): e1000327. 10.1371/journal.pgen.1000327 PMC2621351 19180184

