# Supplementary material for: The DNA Copy Number of Human Endogenous Retrovirus-W (MSRV-Type) Is Increased in Multiple Sclerosis Patients and Is Influenced by Gender and Disease Severity
Source: PLoS One. 2013 Jan 7;8(1):e53623. doi: 10.1371/journal.pone.0053623 (PMC3538585; doi:10.1371/journal.pone.0053623)
Supplement: Figure S1 — Standard Curve of MSRV env made of serial dilutions of genomic DNA. (PDF) [file pone.0053623.s001.pdf]

14 Hilly Street Mortlake NSW 2137 Australia
T + 61 2 9736 1320
F + 61 2 9736 1364
W www.corbettlifescience.com

Quantitation Report

Experiment Information

Run Name	Standard Curves on Genomic DNA_MSRV
Run Start	04/07/2012 11:46:34
Run Finish	04/07/2012 13:28:58
Operator	Marta García-Montojo
Notes	Standard Curve on genomic DNA_MSRV
Run On Software Version	Rotor-Gene 6.1.93
Run Signature	The Run Signature is valid.
Gain FAM	5,

Quantitation Information

Threshold	0,024
Left Threshold	1,000
Standard Curve Imported	No
Standard Curve (1)	conc= 10^(-0,309*CT + 10,286)
Standard Curve (2)	CT = -3,241*log(conc) + 33,341
Reaction efficiency (*)	1,03471 (* = 10^(-1/m) - 1)
M	-3,24148
B	33,34084
R Value	0,99786
R^2 Value	0,99573
Start normalising from cycle	1
Noise Slope Correction	No
No Template Control Threshold	0%
Reaction Efficiency Threshold	Disabled
Normalisation Method	Dynamic Tube Normalisation
Digital Filter	Light
Sample Page	Page 1
Imported Analysis Settings	

Messages

Message

Profile

Cycle	Cycle Point
Hold @ 95℃, 15 min 0 secs	
Cycling (40 repeats)	Step 1 @ 95℃, hold 15 secs
	Step 2 @ 60℃, hold 60 secs, acquiring to Cycling A(FAM)

Raw Data For Cycling A.FAM

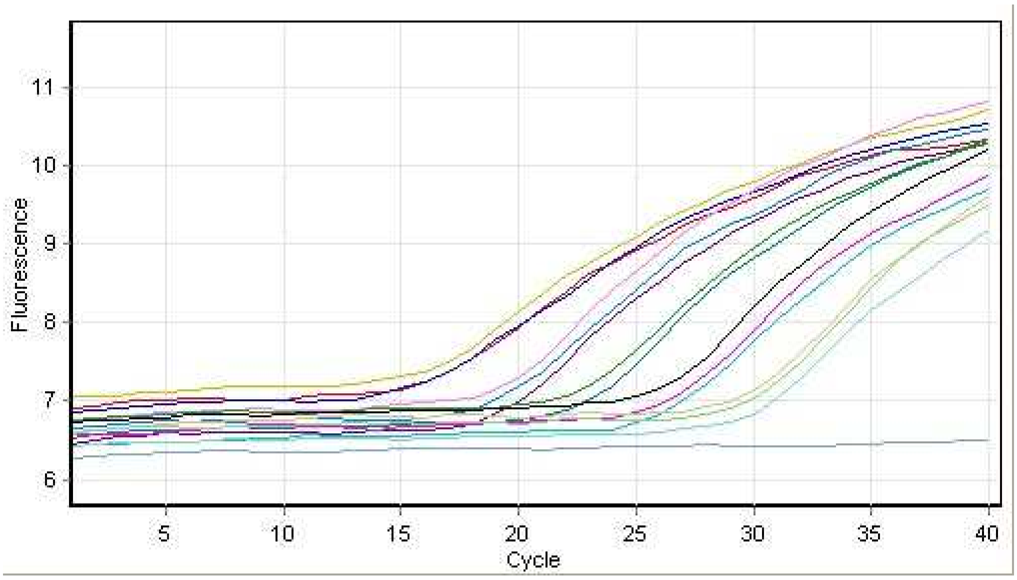

Quantitation data for Cycling A.FAM

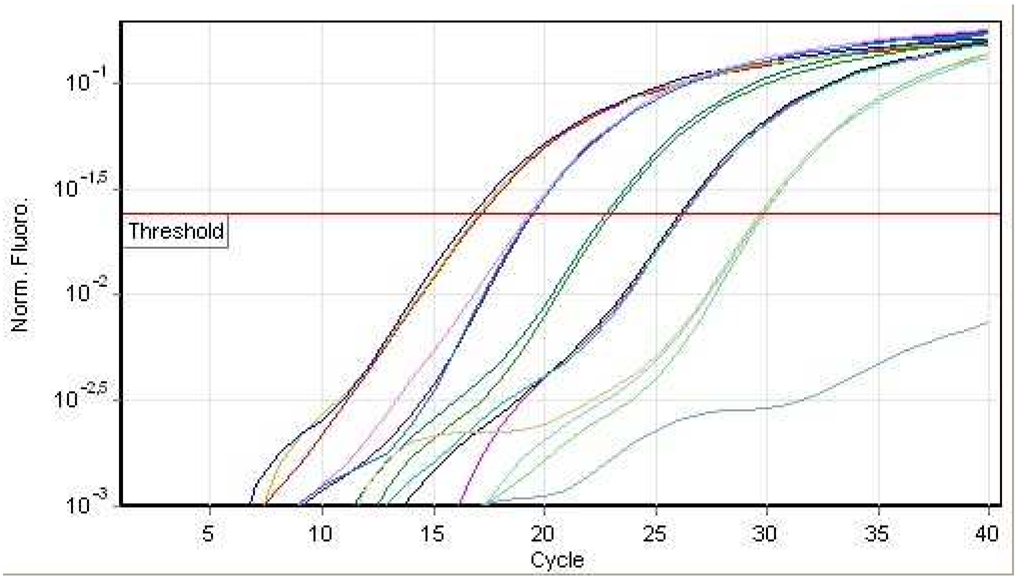

Standard Curve

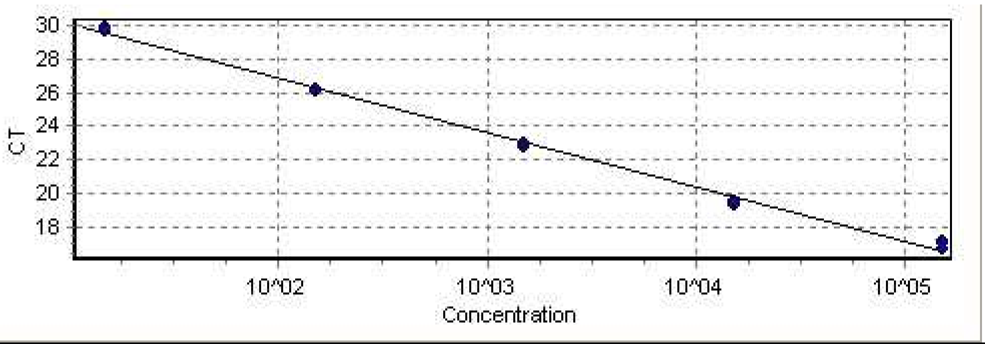

No.	Colour	Name	Type	Ct	Given Conc (Copies)	Calc Conc (Copies)	% Var
A1		Gen DNA 5e5 pg MSRV	Standard	17,11	151.151,0000	101.999,6854	32,5 %
A2		Gen DNA 5e5 pg MSRV	Standard	16,99	151.151,0000	111.092,1604	26,5 %
A3		Gen DNA 5e5 pg MSRV	Standard	16,72	151.151,0000	133.914,8933	11,4 %
A4		Gen DNA 5e4pg MSRV	Standard	19,48	15.151,0000	18.947,6467	25,1 %
A5		Gen DNA 5e4pg MSRV	Standard	19,31	15.151,0000	21.364,5620	41,0 %
A6		Gen DNA 5e4pg MSRV	Standard	19,44	15.151,0000	19.483,7264	28,6 %
A7		Gen DNA 5e3 pg MSRV	Standard	22,74	1.515,0000	1.866,9435	23,2 %
B1		Gen DNA 5e3 pg MSRV	Standard	22,93	1.515,0000	1.632,1140	7,7%
B2		Gen DNA 5e2 pg MSRV	Standard	26,22	151,0000	157,5848	4,4%
B3		Gen DNA 5e2 pg MSRV	Standard	26,06	151,0000	176,7868	17,1 %
B4		Gen DNA 5e2 pg MSRV	Standard	26,19	151,0000	160,8149	6,5%
B5		Gen DNA 5e1 pg MSRV	Standard	29,68	15,0000	13,4598	10,3 %
B6		Gen DNA 5e1 pg MSRV	Standard	29,87	15,0000	11,7370	21,8 %
B7		Gen DNA 5e1 pg MSRV	Standard	29,80	15,0000	12,4105	17,3 %
B8		Neg Control	Negative Control				

This report generated by Rotor-Gene Real-Time Analysis Software 6.1 (Build 93)
©Corbett Research 2005
®All Rights Reserved
ISO 9001:2000 (Reg. No. QEC21313)
