# Supplementary material for: The DNA Copy Number of Human Endogenous Retrovirus-W (MSRV-Type) Is Increased in Multiple Sclerosis Patients and Is Influenced by Gender and Disease Severity
Source: PLoS One. 2013 Jan 7;8(1):e53623. doi: 10.1371/journal.pone.0053623 (PMC3538585; doi:10.1371/journal.pone.0053623)
Supplement: Figure S2 — Standard Curve of RNAse P made of serial dilutions of genomic DNA. (PDF) [file pone.0053623.s002.pdf]

corbett RESEARCH
A Division of Corbett Life Science
14 Hilly Street Mortlake NSW 2137 Australia
T + 61 2 9736 1320
F + 61 2 9736 1364
W www.corbettlifescience.com

Quantitation Report

Experiment Information

Run Name	Standard Curves on Genomic RNAse P
Run Start	04/07/2012 11:46:34
Run Finish	04/07/2012 13:28:58
Operator	Marta García-Montojo
Notes	Standard Curve on genomic DNA_RNase P
Run On Software Version	Rotor-Gene 6.1.93
Run Signature	The Run Signature is valid.
Gain FAM	5,

Quantitation Information

Threshold	0,024
Left Threshold	1,000
Standard Curve Imported	No
Standard Curve (1)	conc= 10^(-0,294*CT + 10,195)
Standard Curve (2)	CT = -3,406*log(conc) + 34,724
Reaction efficiency (*)	0,96604 (* = 10^(-1/m) - 1)
M	-3,4061
B	34,72396
R Value	0,99975
R^2 Value	0,9995
Start normalising from cycle	1
Noise Slope Correction	No
No Template Control Threshold	0%
Reaction Efficiency Threshold	Disabled
Normalisation Method	Dynamic Tube Normalisation
Digital Filter	Light
Sample Page	Page 1
Imported Analysis Settings	

Messages

Message

Profile

Cycle	Cycle Point
Hold @ 95℃, 15 min 0 secs	
Cycling (40 repeats)	Step 1 @ 95℃, hold 15 secs
	Step 2 @ 60℃, hold 60 secs, acquiring to Cycling A(FAM)

Raw Data For Cycling A.FAM

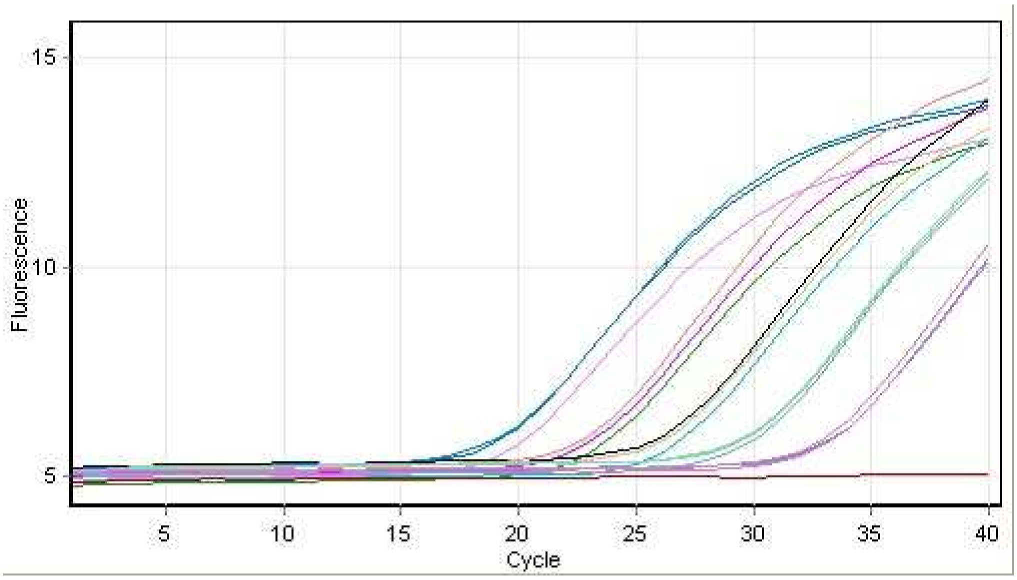

Quantitation data for Cycling A.FAM

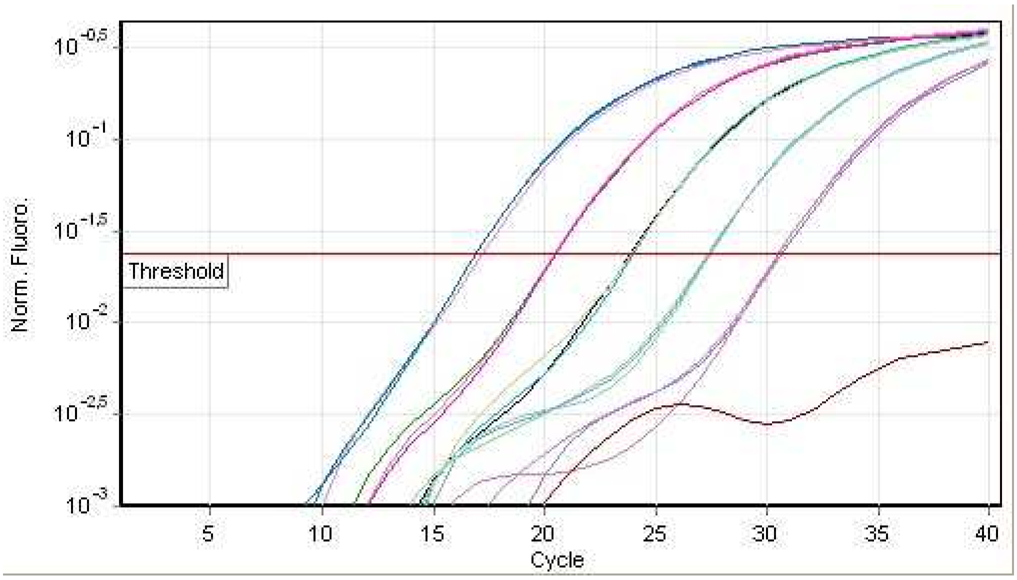

Standard Curve

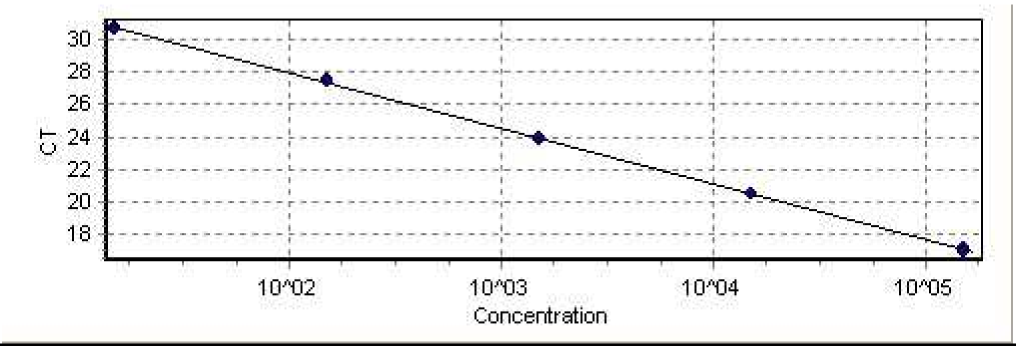

No.	Colour	Name	Type	Ct	Given Conc (Copies)	Calc Conc (Copies)	% Var
E5		Gen DNA 5e5 pg RNAse P	Standard	17,21	151.151,0000	138.851,3290	8,1%
E6		Gen DNA 5e5 pg RNAse P	Standard	16,93	151.151,0000	167.212,1940	10,6 %
E7		Gen DNA 5e5 pg RNAse P	Standard	16,93	151.151,0000	168.021,3672	11,2 %
E8		Gen DNA 5e4 pg RNAse P	Standard	20,48	15.151,0000	15.228,9826	0,5%
F1		Gen DNA 5e4 pg RNAse P	Standard	20,54	15.151,0000	14.646,1550	3,3%
F2		Gen DNA 5e4 pg RNAse P	Standard	20,53	15.151,0000	14.669,7422	3,2%
F3		Gen DNA 5e3 pg RNAse P	Standard	23,85	1.515,0000	1.554,8648	2,6%
F4		Gen DNA 5e3 pg RNAse P	Standard	23,97	1.515,0000	1.436,9732	5,2%
F5		Gen DNA 5e3 pg RNAse P	Standard	23,89	1.515,0000	1.513,5152	0,1%
F6		Gen DNA 5e2 pg RNAse P	Standard	27,38	151,0000	143,4453	5,0%
F7		Gen DNA 5e2 pg RNAse P	Standard	27,48	151,0000	133,8553	11,4 %
F8		Gen DNA 5e2 pg RNAse P	Standard	27,41	151,0000	139,9117	7,3%
G1		Gen DNA 5e1 pg RNAse P	Standard	30,71	15,0000	15,0943	0,6%
G2		Gen DNA 5e1 pg RNAse P	Standard	30,55	15,0000	16,7653	11,8 %
G3		Gen DNA 5e1 pg RNAse P	Standard	30,57	15,0000	16,5376	10,3 %
G4		Neg Control	Negative Control				

This report generated by Rotor-Gene Real-Time Analysis Software 6.1 (Build 93)
©Corbett Research 2005
®All Rights Reserved
ISO 9001:2000 (Reg. No. QEC21313)
