# Supplementary material for: The DNA Copy Number of Human Endogenous Retrovirus-W (MSRV-Type) Is Increased in Multiple Sclerosis Patients and Is Influenced by Gender and Disease Severity
Source: PLoS One. 2013 Jan 7;8(1):e53623. doi: 10.1371/journal.pone.0053623 (PMC3538585; doi:10.1371/journal.pone.0053623)
Supplement: Figure S3 — Standard Curve of MSRV env made of serial dilutions of MSRV env plasmid. (PDF) [file pone.0053623.s003.pdf]

corbett RESEARCH
A Division of Corbett Life Science

14 Hilly Street Mortlake NSW 2137 Australia
T +61 2 9736 1320
F +61 2 9736 1364
W www.corbettlifescience.com

Quantitation Report

Experiment Information

Run Name	Plate 1
Run Start	14/06/2011 13:10:26
Run Finish	14/06/2011 14:55:38
Operator	Marta García-Montojo
Notes	Standard Curve MSRV plasmid
Run On Software Version	Rotor-Gene 6.1.93
Run Signature	The Run Signature is valid.
Gain FAM	5,

Quantitation Information

Threshold	0,024
Left Threshold	1,000
Standard Curve Imported	No
Standard Curve (1)	conc= 10^(-0,310*CT + 12,785)
Standard Curve (2)	CT = -3,230*log(conc) + 41,294
Reaction efficiency (*)	1,03994 (* = 10^(-1/m) - 1)
M	-3,2298
B	41,29422
R Value	0,99836
R^2 Value	0,99673
Start normalising from cycle	1
Noise Slope Correction	No
No Template Control Threshold	0%
Reaction Efficiency Threshold	Disabled
Normalisation Method	Dynamic Tube Normalisation
Digital Filter	Light
Sample Page	Page 1
Imported Analysis Settings	

Messages

Message

Profile

Cycle	Cycle Point

Hold @ 95℃, 15 min 0 se cs	
Cycling (40 repeats)	Step 1 @ 95℃, hold 15 secs
	Step 2 @ 60℃, hold 60 secs, acquiring to Cycling A(FAM)

Raw Data For Cycling A.FAM

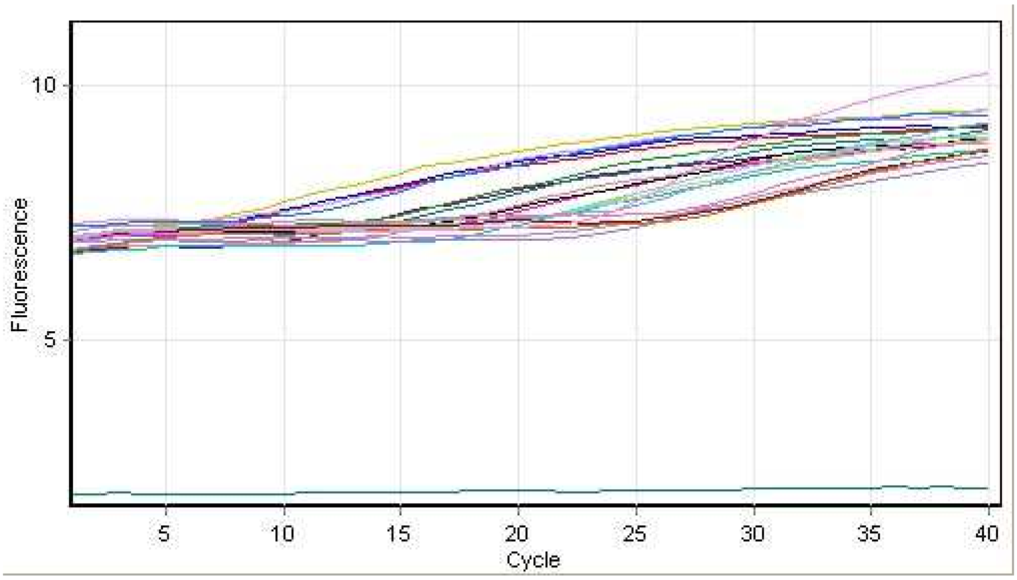

Quantitation data for Cycling A.FAM

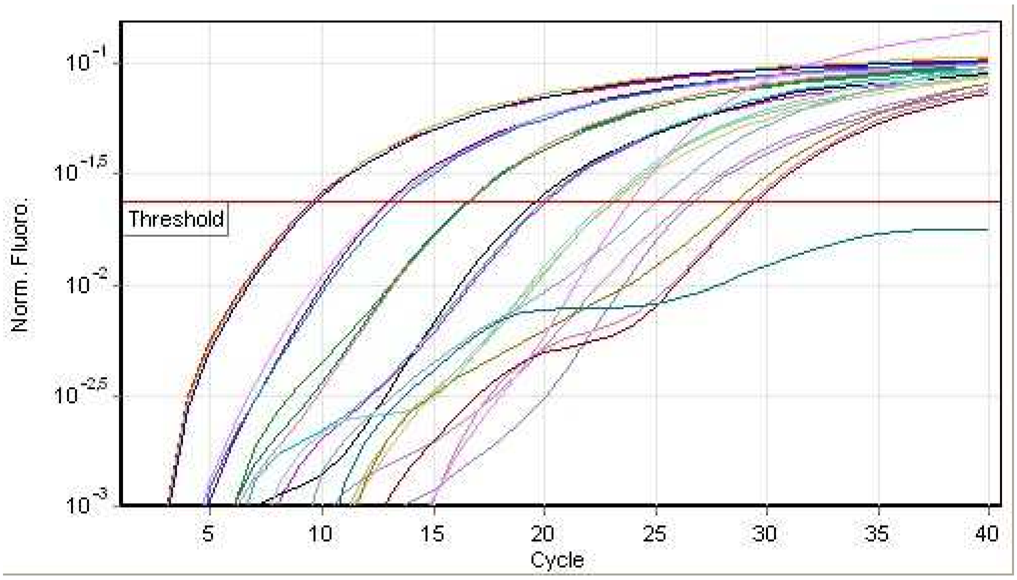

Standard Curve

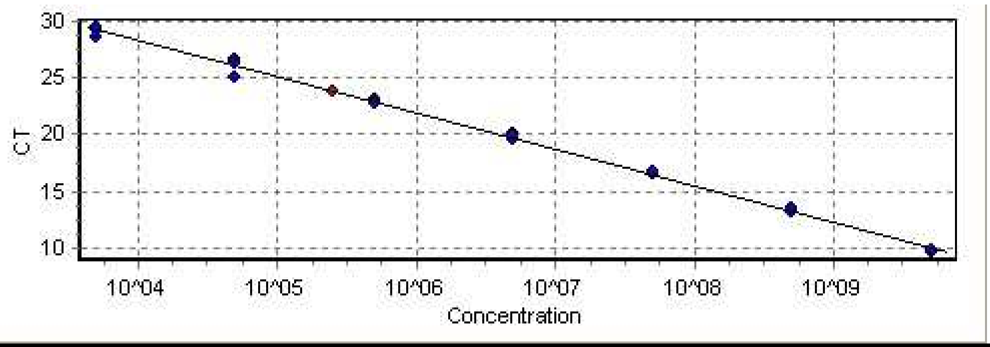

No.	Colour	Name	Type	Ct	Given Conc (Copies)	Calc Conc (Copies)
A1		MSRV 10e9	Standard	9,58	5.000.000.000,0000	6.589.952.220,2125
A2		MSRV 10e9	Standard	9,73	5.000.000.000,0000	5.921.763.119,3230
A3		MSRV 10e9	Standard	9,76	5.000.000.000,0000	5.802.390.937,2535
A4		MSRV 10e8	Standard	13,07	500.000.000,0000	549.425.493,2024
A5		MSRV 10e8	Standard	13,16	500.000.000,0000	513.367.838,5819
A6		MSRV 10e8	Standard	13,49	500.000.000,0000	404.807.113,4418
A7		MSRV 10e7	Standard	16,51	50.000.000,0000	47.308.417,2416
A8		MSRV 10e7	Standard	16,59	50.000.000,0000	44.618.173,6936
B1		MSRV 10e7	Standard	16,62	50.000.000,0000	43.607.605,4318
B2		MSRV 10e6	Standard	20,11	5.000.000,0000	3.629.541,5851
B3		MSRV 10e6	Standard	19,64	5.000.000,0000	5.070.391,7037
B4		MSRV 10e6	Standard	19,97	5.000.000,0000	4.011.760,4879
B5		MSRV 10e5	Standard	23,15	500.000,0000	414.916,7917
B6		MSRV 10e5	Standard	23,02	500.000,0000	456.281,8776
B7		MSRV 10e5	Standard	22,81	500.000,0000	527.082,4269
B8		MSRV 10e4	Standard	25,07	50.000,0000	105.487,0147
C1		MSRV 10e4	Standard	26,72	50.000,0000	32.598,1100
C2		MSRV 10e4	Standard	26,37	50.000,0000	41.905,3019
C3		MSRV 10e3	Standard	29,31	5.000,0000	5.144,3720
C4		MSRV 10e3	Standard	29,5	5.000,0000	4.400,7634

No.	Colour	Name	Type	Ct	Given Conc (Copies)	Calc Conc (Copies)
				3		
C5		MSRV 10e3	Standard	28,63	5.000,0000	8.337,0024
E5		I.S MSRV	Unknown	23,85		251.504,0084
E7		Neg Control MSRV	Negative Control			

This report generated by Rotor-Gene Real-Time Analysis Software 6.1 (Build 93)
©Corbett Research 2005
®All Rights Reserved
ISO 9001:2000 (Reg. No. QEC21313)
